# HDL Is Not Dead Yet

**DOI:** 10.3390/biomedicines10010128

**Published:** 2022-01-07

**Authors:** Shuhui Wang Lorkowski, Jonathan D. Smith

**Affiliations:** 1 Department of Cardiovascular and Metabolic Sciences, Cleveland Clinic, Cleveland, OH 44195, USA; lorkows@ccf.org; 2 Department of Cardiovascular Medicine, Cleveland Clinic, Cleveland, OH 44195, USA

**Keywords:** HDL function, reverse cholesterol transport, apoA1 exchange rate, Mendelian randomization, inflammation, coronary heart disease

## Abstract

High-density lipoprotein cholesterol (HDL-C) levels are inversely correlated with coronary heart disease (CHD) in multiple epidemiological studies, but whether HDL is causal or merely associated with CHD is unclear. Recent trials for HDL-raising drugs were either not effective in reducing CHD events or, if beneficial in reducing CHD events, were not conclusive as the findings could be attributed to the drugs’ LDL-reducing activity. Furthermore, the first large Mendelian randomization study did not causally relate HDL-C levels to decreased CHD. Thus, the hypothesis that HDL is protective against CHD has been rightfully challenged. However, subsequent Mendelian randomization studies found HDL characteristics that are causally related to decreased CHD. Many aspects of HDL structure and function, especially in reverse cholesterol transport, may be better indicators of HDL’s protective activity than simply measuring HDL-C. Cholesterol efflux capacity is associated with lower levels of prevalent and incident CHD, even after adjustment for HDL-C and apolipoprotein A-1 levels. Also, subjects with very high levels of HDL-C, including those with rare mutations that disrupt hepatic HDL uptake and reverse cholesterol transport, may be at higher risk for CHD than those with moderate levels. We describe here several cell-based and cell-free in vitro assays of HDL structure and function that may be used in clinical studies to determine which of HDL’s functions are best associated with protection against CHD. We conclude that the HDL hypothesis may need revision based on studies of HDL structure and function, but that the HDL hypothesis is not dead yet.

## 1. Introduction

HDL has been a popular research topic since the 1970s due to epidemiological studies, such as the Framingham study and many others, that demonstrated the inverse correlation between high-density lipoprotein cholesterol (HDL-C) levels and the incidence or prevalence of coronary heart disease (CHD) [1,2]. HDL is formed by lipid-poor apolipoprotein A-I (apoA1) interacting with the cellular integral membrane protein adenosine triphosphate-binding cassette (ABC) transporter ABCA1, which assembles cellular phospholipids and cholesterol onto the apoA1 scaffold, leading to the production of nascent discoidal HDL [3]. Nascent HDL can accept more cholesterol through the activity of ABCG1, scavenger receptor BI (SR-BI), and aqueous diffusion [4,5]. Spherical mature HDL is formed by the activity of plasma lecithin-cholesterol acyltransferase (LCAT) as it esterifies free cholesterol into cholesterol ester (CE), which, along with some triglycerides (TGs), makes up the hydrophobic core of mature HDL. HDL can then deliver its cholesterol cargo to the liver or other tissues via its major receptor, SR-BI. Alternatively, cholesterol ester transfer protein (CETP) can transfer HDL-CE to apolipoprotein B (apoB)-containing particles, primarily very low-density lipoprotein (VLDL) and intermediate-density lipoprotein (IDL), which in turn can be taken up directly by hepatic remnant receptors or matured into low-density lipoprotein (LDL) and taken up by hepatic LDL receptors. The HDL-mediated delivery of cholesterol from the periphery to the liver for subsequent excretion as free cholesterol or after conversion to bile acids is called the reverse cholesterol transport (RCT) pathway. HDL has been attributed with many different protective effects and pathways in addition to the abovementioned RCT, including cholesterol delivery to steroidogenic organs, anti-inflammatory effects on endothelial cells and macrophages, anti-apoptotic effects on endothelial cells, antithrombotic effects, re-endotheliazation of injured arteries, and anti-oxidant activity [6,7]. Indeed, in hyperlipidemic mouse atherosclerosis studies, overexpression of transgenic human apoA1 led to increases in HDL-C and RCT and decreases in the extent of atherosclerosis [8,9]. Similarly, the RCT effect on reducing atherosclerosis in mice was proven via bone marrow transplantation studies using wild-type or ABCA1-deficient donors. Macrophages, such as those in atherosclerotic lesions, from the ABCA1-deficient donors could not generate nascent HDL from apoA1, and these mice had larger lesions [10]. Thus, HDL-C was considered the “good cholesterol”, and it was hypothesized that the epidemiological association of high HDL-C with less CHD was because HDL was causally protective against atherosclerosis in humans.

## 2. Early Clinical Trials of HDL-Raising Drugs

Although the mouse studies confirmed the anti-atherosclerosis effects of HDL and the RCT pathway, human studies have been controversial. A series of early clinical trials seemed to support the hypothesis that HDL directly protects against CHD. The Coronary Drug Project secondary prevention study of niacin in the 1970s showed that this HDL-raising drug was associated with decreased non-fatal myocardial infarctions compared to placebo [11], but this study might not meet the rigor of current design criteria. Starting in the 1980s, a series of studies were performed using HDL-raising PPARα agonists, such as gemfibrozil. The Helsinki Heart Study was a placebo-controlled double-blinded primary prevention trial of dyslipidemic men followed for five years [12]. Gemfibozil increased HDL-C by 10% and lowered TGs by 43%, and it lowered fatal and non-fatal myocardial infarctions by 34% [12]. The VA-HIT trial was a double-blinded, placebo-controlled gemfibrozil secondary prevention study of men with low HDL followed for five years [13]. HDL-C increased in the treated group by 6% and TGs were lowered by 31%, and there was a significant 24% lower incidence of non-fatal myocardial infarction, stroke, or CHD death [13]. A subsequent subgroup analysis of the VA-HIT trial showed that the significant reduction in events was only in the diabetic subjects; furthermore, the non-diabetic subjects with insulin resistance benefited more from the treatment than those with normal insulin sensitivity [14]. Gemfibrozil and other related PPARα agonists are still in use today. One criticism of these studies is that it is hard to attribute CHD event reductions to increases in HDL-C vs. decreases in non-HDL-C and TGs.

## 3. Recent Clinical Trials of HDL-Raising Drugs

More recent outcomes studies of HDL-C-raising drugs have been performed using niacin [15,16] and CETP inhibitors [17]; however, these concluded with overall discouraging results. The AIM-High study tested niacin effects on outcomes in subjects with low HDL-C. An interim analysis in 2011 halted the study early after the treatment arm showed no signs of additional benefits over placebo in patients already at target levels of LDL, and there was also a small unexplained increase in ischemic strokes [15]. Critics thought the trial was underpowered and poorly designed; even the placebo group took niacin to induce facial flushing such that HDL increased by 25% in the treated arm and 13% in the placebo arm [18]. AIM-High was designed to show a 25% reduction in CHD events but would have needed at least 15 to 20 years of follow up to find this large effect. The HSP2-THRIVE was a large outcomes study testing extended-released niacin and laropiprant, an anti-facial flushing drug, vs. placebo. HDL-C increased by ~16%, while LDL-C and TGs decreased by ~20% in the treated subjects, but 25% of these subjects discontinued due to side effects, including myopathy, which was more common in the statin-using treated subjects [16]. The CHD outcomes were not reported in a full manuscript, but a presentation during the American College of Cardiology meeting reported no apparent reduction in CHD events in the treated arm [18]. Both of these niacin studies were in subjects treated to aggressively low LDL-C levels, and thus event rates may not have been high enough to see beneficial effects of niacin [18].

Four different CETP inhibitors were tested on top of statins or other LDL-lowering drugs in large outcomes trials. Torceptrapib raised HDL-C by 72%, but it led to a significant increase in blood pressure, and the trial was halted early after a 50% increase in deaths in the treated group [19]. Dalcetrapib increased HDL-C by 36% with a small increase in blood pressure, but the trial was stopped early due to futility [20]. The evacetrapib trial was also halted early due to futility [21]. An outcomes trial with ~30,000 patients followed for ~4 years was performed for anacetrapib [22]. The treated group showed a >100% increase in HDL-C and a 17% reduction in non-HDL-C. The treated group demonstrated a ~10% significant reduction in myocardial infarctions, CHD death, and coronary revascularization procedures [22]. Again, the benefits of treatment might have been due to the lowering of non-HDL cholesterol and the size and duration of the trial; in addition, anacetrapib accumulated in fat tissue, leading to discontinuation of plans for its marketing. In conclusion, modern drug trials do not fully support the HDL hypothesis that more HDL-C is protective against CHD.

Due to the inverse association of HDL-C with CHD, and the proven effect of apoA1 overexpression decreasing atherosclerosis progression in mouse models, apoA1, reconstituted HDL (rHDL), or apoA1 mimetic peptides have been utilized in multiple pre-clinical and clinical trials. An early clinical trial used apoA1 Milano, a missense variant that was thought to have a slight gain of function, formulated in rHDL; after four weekly treatments there was a small, but significant, decrease in coronary artery plaque volume, determined by intravascular ultrasound [23]. However, a more recent trial of a different apoA1 Milano rHDL formulation did not show any benefit in lesion regression in acute coronary syndrome patients [24]. After infusion of a wild-type apoA1 rHDL therapy (CSL112), massive remodeling of HDL occurred with increases in two small and one large HDL species; increased cholesterol efflux capacity in the apoB-depleted serum was also observed [25,26]. There are several ongoing CHD event trials using various rHDL or HDL mimetics, which have been reviewed recently [27].

## 4. Genetic Studies of HDL Causality in CHD Prevention

Genetic studies of rare and common variants in or near genes affecting HDL-C have been assessed in several population studies in order to determine if they also alter CHD risk, resulting in inconsistent findings. Rare heterozygous carriers of ABCA1 loss-of-function mutations in three Danish cohorts were found to have lower HDL-C levels but did not have increased risk of CHD [28], which is not in agreement with the HDL hypothesis. Subjects who were carriers of a rare loss-of-function variant (P376L) in the SR-BI gene (*SCARB1* is official gene symbol) showed significantly increased HDL-C levels and an increased risk of CHD [29], which is again not supportive of the HDL hypothesis. Similarly, a more common (4% allele frequency) missense variant (S208T) in the *LCAT* gene is associated with decreased HDL-C but not with increased risk of CHD [30]. In contrast, two common SNPs leading to reductions in CETP activity and increased HDL-C levels are also associated with decreased risk of CHD [31], thus supporting the HDL hypothesis.

Instead of studying one gene at a time, more recent genome-wide association studies (GWAS) have identified hundreds of loci harboring common SNPs affecting plasma lipid and lipoprotein levels; however, many of these loci are pleiotropic, affecting more than one lipid traits [32]. To determine if the constellation of common SNPs associated with HDL-C are causal for CHD, a genetic test called Mendelian randomization is used, where HDL-associated SNPs are tested for association with CHD in the predicted direction and effect size. The first large Mendelian randomization study for HDL and CHD did not show causality; although, due to the pleiotropic effects of many SNPs this study was limited to variants that only affect HDL-C and not LDL-C or TGs also [33]. This study was perhaps more influential in disproving the HDL hypothesis then even the drug trials. However, three more recent Mendelian randomization studies have resurrected the HDL hypothesis. First, Tall and colleagues demonstrated that HDL-C levels are independently associated with CHD after multivariable Mendelian randomization that adjusted for pleiotropic effects on lipid traits and related traits including body mass index, type 2 diabetes, and blood pressure [34]. Second, Elosua and colleagues identified GWAS SNPs affecting HDL-C, apoA1, HDL size, particle number, and lipid content, and then used these SNPs in a multivariate Mendelian randomization study after adjusting for pleiotropy. This study found only certain HDL traits, such as HDL-C, in medium-sized HDL and the number of very large HDL particles was inversely related to CHD risk [35]. Third, Zhao, Rader, and colleagues performed a Mendelian randomization using SNPs associated with the size, particle number, and lipid composition of VLDL, LDL, and HDL. After multivariate adjustment, they found that variants associated with the levels and composition of medium and small HDL particles have protective effects against CHD [36]. Still, compared to the straightforward causal relationships of LDL-C and TGs for CHD, many complicated methods are required to find HDL traits that are causally protective against CHD. These studies suggest that it is not simply HDL-C that is protective, and they provide more reasons to look at HDL structure and functions rather than just HDL-C levels.

## 5. Very High HDL-C May Not Be Good

Early evidence of a possible threshold effect of HDL-C and CHD risk came from a Norwegian study which enrolled >47,000 middle-aged men and women. This study found a U-shaped curve for association of HDL-C levels with CHD and all-cause mortality, such that there were more events when HDL-C was > 58 mg/dL in men [37]. A post hoc analysis of two prospective studies demonstrated that both higher HDL-C and larger HDL particles, after adjustment for apoA1 and apoB levels, are associated with increased major cardiac event risk factors [38]. In six community-based clinical cohorts with >11,000 men and women, each followed on average for more than 12 years, CHD risk was found to be inversely associated with HDL-C up to a point, but there was no further reduction in CHD risk with HDL-C values higher than 90 mg/dL in men and 75 mg/dL in women [39]. In a study of 631,000 Canadians followed on average for ~5 years, the U-shaped association was again observed, as both male and female individuals with low HDL-C had increased all-cause mortality compared to those with average HDL-C levels; however, there was also increased all-cause mortality for men with HDL-C > 70 mg/dL and women with HDL-C > 90 mg/dL [40]. A study of two prospective Scandinavian cohorts found that the association between HDL-C levels and all-cause mortality was U-shaped in both men and women, with both high and low HDL-C associated with higher mortality, even after multifactorial adjustment [41]. A similar conclusion has been reached in a study involving 1.7 million US veterans followed for nine years that evaluated the relationship between HDL-C and risk of death [42] and also in a large general population study with 37,059 participants [43]. This U-shaped association of HDL-C with CHD mortality was also found in a large Japanese study, with high HDL-C associated with significantly more events in men, and a similar, but not significant, trend in women [44]. Similar findings were reported in four additional Asian studies [45,46,47,48].

These epidemiological studies cannot provide a mechanism explaining why very high HDL-C leads to increased risk. It has been suggested that this U-shaped association may be due to: (1) genetic variants that lead to high HDL and high CHD risk; (2) confounding factors associated with increased mortality and high HDL (e.g., alcohol intake); or (3) the accumulation of dysfunctional HDL that may increase CHD risk [49]. One example of a genetic cause is the rare SR-BI mutation, mentioned above, associated with increased HDL-C and CHD risk. Functional studies of the mutant SR-BI protein in both transfected cells and in induced pluripotent stem cell-derived hepatocytes showed that the mutant protein has decreased selective HDL cholesterol ester uptake and that the wild type but not the mutant protein can lower HDL-C levels in SR-BI knockout mice [29]. Hepatic SR-BI delivers HDL-C to the liver and SR-BI knockout mice have been shown to have decreased macrophage-to-feces RCT [50]. Thus, if HDL is high, but cannot contribute to RCT for whatever reason, it may actually become an atherosclerosis-promoting lipoprotein, as evidenced by more severe atherosclerosis in apoE/SR-BI double knockout vs. apoE knockout mice [51]. Thus, HDL-C may not be protective against CHD and it may be the functions of HDL, such as in RCT and its antioxidant, anti-inflammatory, and anti-thrombotic activities, that are protective. Additional mechanistic studies will be required in order to determine the reason for the U-shaped association between HDL-C and all-cause mortality.

## 6. Cell-Based HDL Function Assays

### 6.1. Cholesterol Efflux Capacity

**Figure 1:**
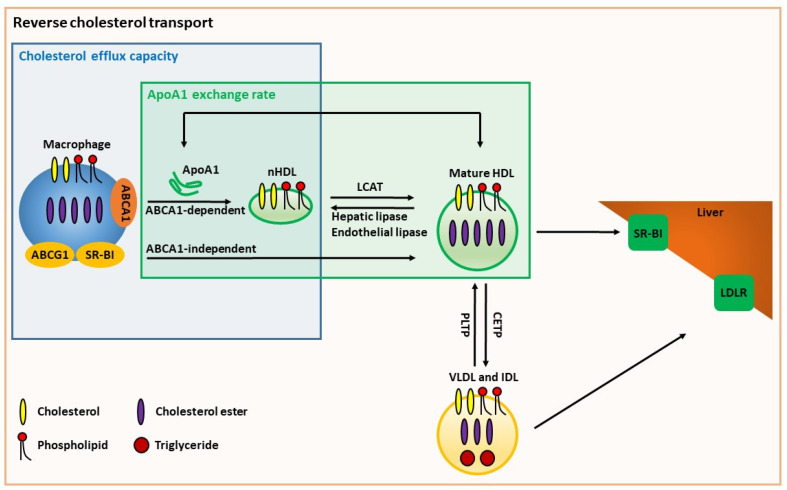
Assays for HDL function that represent the role of HDL in reverse cholesterol transport and HDL remodeling. The cholesterol efflux capacity assay uses human apoB-depleted serum as an acceptor for macrophage cholesterol and phospholipids. ABCA1 mediates cellular cholesterol and phospholipid efflux onto serum lipid-poor apoA1 leading to the formation of nHDL, which represents the ABCA1-dependent cholesterol efflux capacity. Serum HDL can accept more cholesterol and phospholipid through ABCG1, SR-BI, and aqueous diffusion, which represents the ABCA1-independent cholesterol efflux capacity. This assay can distinguish between ABCA1-dependent and -independent cholesterol efflux when using mouse macrophages as the donor cell type. The apoA1 exchange-rate assay is a cell-free in vitro assay in which human apoB-depleted serum or plasma is incubated with exogenously labeled apoA1, and apoA1 is freely exchangeable between labeled lipid-free apoA1 and serum HDL. The rate of exchange of the labeled apoA1 is an indicator of HDL remodeling and is correlated with plasma HDL and apoA1, as well as with the cholesterol efflux capacity. ABCA1: ATP-binding cassette transporter A1; ABCG1: ATP-binding cassette transporter G1; SR-BI: scavenger receptor B I; ApoA1: apolipoprotein A-I; nHDL: nascent high-density lipoprotein; LCAT: lecithin:cholesterol acyltransferase; CETP: cholesteryl ester transfer protein; PLTP: phospholipid transfer protein; VLDL: very low-density lipoprotein; IDL: intermediate-density lipoprotein; LDLR: low-density lipoprotein receptor.

The role of HDL in RCT may be one of its most important atherosclerosis-protective functions. The first step of this pathway occurs when lipid-poor apoA1, the major lipoprotein in HDL, is assembled with phospholipids and cholesterol from peripheral cells into nascent HDL via the membrane protein ABCA1. Additional cholesterol can be effluxed from cells to HDL via ABCG1 and other pathways. The function of HDL in this part of the RCT pathway can be estimated via the cholesterol efflux capacity (CEC) assay, in which apoB-depleted human serum from different donors is used as the cholesterol acceptor (Figure 1). In this assay, typically mouse macrophage cell lines (usually RAW264.7 or J774) are labeled with ^3^H-cholesterol [52,53,54,55] or Bodipy-cholesterol [56] overnight, treated with or without cyclic adenosine monophosphate (cAMP) analogues to induce ABCA1 expression in cells [57] for 16 h and chased with apoB-depleted serum for 4 h. Basal CEC (without cAMP) represents ABCA1-independent CEC, while total CEC (with cAMP) represents ABCA1-dependent and -independent CEC. Thus, ABCA1-dependent CEC is calculated by subtracting the basal CEC from the total CEC. Human THP-1 macrophages may also be used, but ABCA1 is expressed basally and cAMP does not induce human ABCA1 (although LXR agonists may be used); thus, one cannot fully dissect the ABCA1-dependent and -independent CEC. CEC may capture key HDL traits that are not captured simply by measuring HDL-C. Lipid-poor apoA1 (preβ HDL) averages around 5% of the total apoA1 pool, though it may differ from person to person, and the lipid-poor apoA1 is the preferred substrate for ABCA1-dependent CEC [58]. Additionally, HDL particle concentration determined by ion mobility analysis correlates better with CEC than HDL-C levels [59]. In a pioneering study, Khera et al. showed that decreased total CEC in apoB-depleted serum is associated with prevalent CHD, and in a healthy subject cohort decreased total CEC was correlated with carotid artery intima-media thickness, with these findings remaining significant after adjustment for CHD risk factors and either HDL-C or apoA1 levels [52]. Many subsequent studies have been performed extending the inverse association of CEC with incident major adverse cardiovascular events (MACEs) [54,55,56], as summarized in Table 1. However, one study showed that enhanced total CEC is associated with reduced risk of prevalent CHD, yet paradoxically with increased incident MACEs [53]. Even in healthy young adults, the inverse association of total CEC with subclinical cardiovascular risk markers was confirmed in a much larger study [60]. Most of these large cohort studies did not dissect total CEC into ABCA1-dependent and ABCA1-independent CEC. Recently, by measuring HDL-mediated changes in cholesterol mass in the media of THP-1 differentiated macrophages, an assay called cholesterol mass efflux capacity (CMEC) was assessed without using radioisotope- or fluorescent- labeled cholesterol [61]. Consistent with CEC, CMEC has an inverse relationship with CHD and incident events [61]. One feature of apoA1 and HDL that can decrease cholesterol efflux capacity is their oxidation by myeloperoxidase (MPO). ApoA1 is a selective target for MPO-catalyzed oxidation in plasma, and the binding site for MPO on HDL has been identified [62,63]. Although MPO modifies apoA1 on tyrosine, lysine, and methionine residues, the modification that severely decreases apoA1’s efflux capacity is tryptophan oxidation, which primarily creates the 2-OH-tryptophan that is found on about 1/5 of all HDL molecules isolated from human atheroma [64,65].

### 6.2. Endothelial Protective Actions

HDL also has many protective effects on endothelial cells, including protection against apoptosis, induction of endothelial nitric oxide synthase (eNOS), and reduction of adhesion molecule expression. All of these activities are diminished in MPO-oxidized HDL, which loses its ability to bind to SR-BI expressing cells [66]. Oxidized LDL induces monocyte adhesion to co-cultures of endothelial and smooth muscle cells, which is prevented by the addition of HDL [67]. Endothelial cell incubation with LDL induces LDL oxidation, which is inhibited by co-incubation with HDL [67]. HDL also promotes endothelial migration and proliferation in in vitro scratch-wounded monolayers [68]. In vivo, knockout of either apoA1 or SR-BI leads to delayed re-endothelialization after carotid artery endothelial denudation, demonstrating an important function of HDL [68].

### 6.3. Antithrombotic Activity

HDL has also been demonstrated to have antithrombotic activity [69]. Incubation of isolated platelets with oxidized LDL induces platelet aggregation, increased reactive oxygen species, and biding to neutrophils, all which can be inhibited by pretreatment of the platelets with HDL, in a dose-dependent manner [70].

## 7. In Vivo Assays for HDL Function

Numerous RCT studies have been performed in mice using ^3^H-cholesterol-labeled macrophages injected i.p. or s.c. to follow the radioactivity transfer from the cells to the plasma, liver, bile, and feces. One example demonstrated that s.c. injection of human apoA1, but not MPO-oxidized apoA1, into apoA1-deficient mice increased RCT. Similarly, four injections, given every other day, of apoA1, but not MPO-oxidized apoA1, decreased lesion macrophage and lipid content in apoE-deficient mice [71].

An in vivo method was developed recently to measure macrophage-specific RCT in humans in which subjects receive albumin-bound ^3^H-cholesterol nanoparticles intravenously [72]. In mouse studies, this tracer rapidly disappears from plasma after the injection and is taken up by hepatic Kupffer cells, and it then reappears after efflux into the plasma [72]. This assay has been tested in healthy humans and the reappearance of plasma ^3^H-cholesterol as free and esterified cholesterol was found both in the HDL and non-HDL fractions, the latter presumably obtained via transfer into apoB-lipoproteins by CETP [72].

## 8. HDL Structure and Cell-Free HDL Function Assays

### 8.1. HDL Subpopulations

The CEC assay is a well-recognized assay of HDL function; however, this gold standard assay is hard to transform into a clinical diagnostic assay due to the requirement of cell culture and the time- and labor-intensive time course (usually 4 days). Therefore, several labs have pursued the development of more rapid clinically-relevant assays of HDL structure and cell-free assays of HDL function, as summarized in Table 2. HDL is a heterogeneous group of small discoid and spherical particles that differ in size, density, and electrophoretic mobility, with a mean size of 7–12 nm and density of 1.063–1.21 g/mL [73]. Due to the heterogeneity in HDL size and structure, these subpopulations may be differentially associated with cardiovascular risk. One approach to study HDL heterogeneity involves two-dimensional electrophoresis, which allows the separation by size and charge to resolve 12 distinct HDL subpopulations [74]. The levels of small, lipid-poor preβ-1 and intermediate α-2 HDL correlate well with ABCA1-mediated cholesterol efflux, while the levels of α-2, α-1, α-3, and preβ-1 HDL all correlate well with SR-BI-mediated cholesterol efflux [75]. In clinical studies, the level of α-1 HDL increases after simvastatin-niacin therapy and is associated with decreased progression of coronary stenosis [76]. In VA-HIT, patients with low levels of α-1 and α-2 HDL and high levels of α-3 levels HDL are associated with an increased risk for CHD events [77]. Another approach to study HDL heterogeneity is nuclear magnetic resonance (NMR) spectroscopy, which can determine the number of large, medium, and small HDL particles [78]. The clinical utility of this assay was estimated in a nested case-control study, in which both HDL particle concentration and HDL size are associated with CHD risk [79]. However, the association of HDL particle concentration with CHD was found to be not significant after the adjustment for myeloperoxidase, paraoxonase, and C-reactive protein levels; likewise, the association of HDL size with CHD disappeared after the adjustment for apoB and triglyceride levels [79]. In a large prospective cohort with 27,673 women followed for 11 years, NMR-measured HDL particle concentration and size showed comparable, but not superior, prediction of CHD risk compared to standard lipid levels [80]. In a pooled cohort of four large population studies, NMR-measured HDL particle concentration was found to be inversely associated with myocardial infarction and ischemic stroke [81]. NMR was also used in the recent Mendelian randomization studies, in which NMR-measured HDL characteristics showed significant causal association with CHD risk [35,36].

### 8.2. HDL Proteome

HDL structure has also been assessed by proteomics. Various methods to gently isolate HDL, without centrifugation—such as size exclusion chromatography, anti-apoA1 antibody pulldown, and biotin-labeled apoA1 exchange into HDL with streptavidin pulldown—have been used, followed by mass spectrometry proteomics. HDL acts as a scaffold and can carry many different plasma proteins, with 251 proteins reproducibly associated with HDL, although each HDL particle can only carry a few other proteins. As reviewed by Davidson, six studies identified 109 HDL proteins showing consistent differences between CHD subjects and healthy controls [82]. Jin et al. assessed the HDL proteome after his-tagged-apoA1 exchange and metal chelate affinity chromatography. Proteomic associations with CEC and prevalent CHD were identified that were used to develop multivariate algorithms predictive for CEC and CHD [83]. The predicative CHD model was tested in a case-control cohort, showing an area under the curve value of 0.73 in distinguishing prevalent CHD in receiver operating characteristic analysis [83].

### 8.3. ApoA1 Exchange Assays

Moving beyond HDL structure, functional assays have been developed based on ApoA1′s exchange between lipid-free and lipid-bound states (Figure 1). Cavigiolio et al. showed that fluorescently labeled apoA1 can exchange into rHDL and, vice versa, fluorescently labeled apoA1 made into rHDL can be chased into the lipid-poor fraction by incubation with excess unlabeled apoA1 [84]. Lipid-free apoA1 exchange into serum or plasma-derived human HDL can then be assessed as an indication of HDL remodeling. An HDL-apoA1 exchange end-point assay was developed by incubating spin-labeled lipid-free apoA1 with apoB-depleted plasma at 37 °C for 15 min, with exchange of the apoA1 probe into HDL quantified by electron paramagnetic resonance spectrometry [85,86]. HDL-apoA1-exchange was found to be lower from 16 acute coronary syndrome patients compared to 9 healthy control donors in a small human study, and this exchange was correlated with CEC, HDL-C, and apoA1 levels [85,86]. In addition, HDL-apoA1-exchange was inversely correlated with prevalent atherosclerotic severity measured by angiography in patients with type 2 diabetes [87]. However, HDL-C had a similar inverse correlation; thus, the added utility of this exchange assay was not demonstrated [87].

We synthesized a dual fluorescent-labeled apoA1 lipidation indicator (NBD/Alexa647-apoA1) in which the NBD label is lipid-sensitive while Alexa647 is not; we showed that the NBD/Alexa647 emission ratio increases after incubation with liposomes or cultured cells [88]. We then used this NBD/Alexa647-apoA1 to measure the apoA1 exchange rate (AER) by incubating it with apoB-depleted plasma or serum at 37 °C for 1 h. The NBD/Alexa647 emission ratio of this indicator increases over time upon its exchange into HDL, and the rate of this increase is defined as the AER, an indicator of HDL remodeling and function [89]. In a large prospective cohort, we found that the subjects in the lowest quartile of AER had increased incident MACEs after adjustment for traditional risk factors including HDL-C and apoA1 levels [89]. AER was well-correlated with ABCA1-independent CEC and not with ABCA1-dependent CEC [89]. This result was confirmed in a separate 5-year follow-up bariatric surgery cohort of patients with obesity and type 2 diabetes, in which the change in AER from baseline to year 5 was correlated with the reduction in body mass index and glycated hemoglobin levels [90].

Another cell-free assay for HDL function is its capacity to take up Bodipy-labeled cholesterol from liposomes after incubation with apoB-depleted serum. The amount of Bodipy-labeled cholesterol in the HDL is determined by precipitating with an anti-apoA1 antibody [91]. This cholesterol uptake capacity was well-correlated with total CEC and inversely correlated with the requirement for revascularization in patients with optimal control of LDL choleterol [91]. A similar cell-free cholesterol exchange assay was developed using large multilamellar vesicles containing Bodipy-labeled cholesterol incubated with apoB-depleted serum [92]. The exchange into HDL in the supernatant was measured and was well-correlated with total CEC measured using THP-1 cells [92].

### 8.4. Antioxidant Activity

A well-studied function of HDL is its antioxidant activity, both in cell-based assays (mentioned above) and in cell-free assays. The in vitro copper ion-induced oxidation of LDL is inhibited by HDL, which is mediated by paraoxonase carried on HDL [93,94]. HDL can also inhibit the oxidation of the phospholipid palmitoyl-arachidonoyl phosphatidylcholine (PAPC), which is initiated by the addition of the lipid hydroperoxide [95].

## 9. Cholesterol Efflux and Inflammation

In cholesterol efflux pathways, ABCA1 mediates cholesterol efflux to lipid-free apoA1, while ABCG1 mediates cholesterol efflux to nascent and mature HDL [96]. Increased cellular cholesterol content in macrophages and other immune cells promotes inflammatory responses, and cholesterol efflux pathways mediated by ABCA1 and ABCG1 may have anti-atherogenic effects by suppressing inflammatory responses [97]. Mice deficient in ABCA1 and ABCG1 display leukocytosis, increased proliferation of hematopoietic stem and multipotential progenitor cells in the bone marrow, and accelerated atherosclerosis [98]. ABCA1 and ABCG1 knockout specific to macrophages, and not in hematopoietic stem and multipotential progenitor cells, still leads to monocytosis and neutrophilia and increased atherosclerosis [99]. Similarly, mice deficient in ABCA1 and ABCG1 specifically in endothelial cells demonstrate decreased eNOS activity, increased endothelial inflammation, and accelerated atherosclerosis [100]. The positive outcome from the CANTOS trial, in which the anti-inflammatory therapy canakinumab targeting the innate immunity pathway-product interleukin-1β (IL-1β) reduces recurrent cardiovascular events independent of lipid lowering [101], further confirmed the importance of inflammation in the pathogenesis of atherosclerosis. Cholesterol accumulation in ABCA1- and ABCG1-deficient myeloid cells activates the NLRP3 inflammasome that is required for IL-1β and IL-18 secretion and enhances neutrophil extracellular traps in atherosclerotic plaques [102]. Notably, patients with Tangier disease with loss-of-function ABCA1 mutations have increased levels of IL-1β and IL-18 [102]. It has also been shown that ABCA1 mutation carriers who are not on statins have increased plaque inflammation vs. controls, assessed by positron emission tomography-MRI [103]. In addition, rHDL infusions in *Apoe^−/−^* or *Ldlr^−/−^* atherosclerotic mice produce anti-inflammatory effects in lesion macrophages [104].

## 10. Conclusions

The hypothesis that HDL protects against CHD is still under debate and the subject of further research. What is becoming increasingly clear from genetic and drug studies is that atherosclerosis is driven largely by LDL-C and TGs, and that the case for a protective role for an HDL function is not as straightforward as previously thought. However, we argue that HDL metabolism and function is much more complex than the deposition of LDL-C to the artery wall. Dysfunctional oxidized HDL can be found in human atheroma. There is also a growing body of literature about the U-shaped association of HDL-C with CHD such that both those with the lowest and highest levels of HDL-C have increased risk. Animal models have proven the RCT pathway and that increases in macrophage-to-feces cholesterol delivery, such as in apoA1 transgenic mice, leads to decreased atherosclerosis progression. Clever studies of human HDL structural and functional attributes have found associations with decreased CHD, including newer Mendelian randomization studies that suggest causal effects. Thus, we conclude that the HDL hypothesis is not dead, at least not yet. Future studies of the HDL hypothesis should focus on the various functions and the myriad subclasses of HDL, along with modifications that render HDL dysfunctional or even pro-atherogenic.

## Figures and Tables

**Table 1:** Prospective clinical studies of HDL function assays.

Clinical Study [Reference]	HDL Function Assay	Outcome
Dallas Heart Study [56]	CEC using J774 macrophages and Bodipy-cholesterol	Inverse correlation between CEC and incident cardiovascular events
Epic-Norfolk Study [54]	CEC using J774 macrophages and ^3^H-cholesterol	Inverse correlation between CEC and incident CHD
GeneBank [53]	CEC using RAW macrophages and ^3^H-cholesterol	Positive correlation between CEC and incident MACE
Jupiter rial [55]	CEC using J774 macrophages and ^3^H-cholesterol	Inverse correlation between CEC and incident CVD at statin therapy but not at baseline
MESA study [61]	Cholesterol mass efflux capacity using THP-1 differentiated macrophages	Inverse correlation between cholesterol mass efflux capacity and incident CVD

**Table 2:** Cell-free HDL function assays.

Assay	Methodology	Clinical Relevance	Practicality
NMR spectroscopy	Quantify the number and size of HDLs	Inverse association between HDL-particle concentration and CHD	Requires NMR spectroscopy
ApoA1-associated proteome panel	Generate a multivariate algorithm for CHD prediction through multiprotein analysis	Predictive for CHD	High-throughput, requires mass spectrometry
ApoA1 exchange assay	Quantify spin-labeled lipid-free apoA1 probe exchanged into HDL by electron paramagnetic resonance spectrometry	Inverse association between apoA1 exchange and prevalent CHD in small clinical studies	High-throughput, requires an electron paramagnetic resonance spectrometer
ApoA1 exchange rate	Quantify the rate of NBD/Alexa647-labeled lipid-free apoA1 probe exchanged into HDL	Inverse association between apoA1 exchange rate and incident MACE	High-throughput
Cholesterol uptake capacity	Detect the capacity of HDL to take up Bodipy-labeled cholesterol from liposomes	Inversely associated with the requirement for revascularization in a small clinical study	High-throughput
Cholesterol exchange assay	Quantify Bodipy-labeled cholesterol from liposomes exchanged into HDL	Lacks clinical evidence	High-throughput
Antioxidant assay	Detect the ability of HDL in preventing the formation of oxidized phospholipids	HDLs from CHD patients lose antioxidant function in small clinical samples	High-throughput
